# The Novel Internalins InlP1 and InlP4 and the Internalin-Like Protein InlP3 Enhance the Pathogenicity of *Listeria monocytogenes*

**DOI:** 10.3389/fmicb.2019.01644

**Published:** 2019-07-23

**Authors:** Eva Harter, Caroline Lassnig, Eva Maria Wagner, Andreas Zaiser, Martin Wagner, Kathrin Rychli

**Affiliations:** ^1^Department for Farm Animals and Public Health in Veterinary Medicine, Institute of Food Safety, Food Technology and Veterinary Public Health, University of Veterinary Medicine Vienna, Vienna, Austria; ^2^Department of Biomedical Sciences, Institute of Animal Breeding and Genetics and Biomodels Austria, University of Veterinary Medicine Vienna, Vienna, Austria; ^3^Austrian Competence Centre for Feed and Food Quality, Safety and Innovation – FFoQSI GmbH, Tulln, Austria

**Keywords:** *Listeria monocytogenes*, outbreak strain, accessory genome, virulence, internalin, internalin-like protein

## Abstract

The pathogenicity of the human foodborne pathogen *Listeria monocytogenes* relies on virulence factors such as internalins. In 2009/2010 two *L. monocytogenes* strains were responsible for a serious listeriosis outbreak in Austria, Germany, and the Czech Republic. One of these clones, QOC1, which caused 14 cases including five fatalities, encodes the novel internalins *inlP1*, *inlPq* and *inlP4*, and the novel internalin-like protein *inlP3* in the genomic region of hypervariable genetic hotspot 9 in addition to the standard set of virulence genes. The *in silico* prevalence study revealed that these genes rarely occur in *L. monocytogenes*, mainly in minor clonal complexes. To obtain first insights of the role of these genes in the pathogenicity of *L. monocytogenes*, we studied the gene expression under conditions mimicking the ingestion in the host. Expression of *inlP1*, *inlP3*, *inlPq* and *inlP4* was increased under gastric stress and in intracellular bacteria grown in intestinal epithelial cells. Furthermore, colonization of the liver and the spleen was slightly, but significantly reduced 72 h post infection in an oral mouse infection model when *inlP1* or *inlP4* was deleted. Moreover, the impact of InlP1 and InlP3 in virulence was shown *in vitro* in human intestinal epithelial cells. In this study we conclusively demonstrate a potential contribution of uncommon novel internalins and an internalin-like protein to the pathogenicity of *L. monocytogenes*.

## Introduction

*Listeria monocytogenes* is one of the most concerning human foodborne pathogens as it is the causative agent of listeriosis, a rare but severe disease that is associated with high mortality rates. After ingestion of contaminated food, listeriosis can manifest as a non-invasive self-limiting gastroenteritis in healthy individuals but also as an invasive and systemic infection in immunocompromised and elderly individuals leading to meningoencephalitis or septicemia (Vázquez-Boland et al., 2001). Infection during pregnancy can lead to placentitis, abortion, stillbirth or neonatal infection (Madjunkov et al., 2017). Especially ready-to-eat products are at high risk of contamination with the pathogen over the course of food processing (Gandhi and Chikindas, 2007).

In 2009 and 2010, contaminated Austrian Quargel cheese was responsible for a multinational listeriosis outbreak in Austria, Germany, and the Czechia with 34 cases (Fretz et al., 2010a,b). Two *L. monocytogenes* strains were assigned to this outbreak: Quargel Outbreak Clone 1 (QOC1), which caused 14 cases including five fatalities and Quargel Outbreak Clone 2 (QOC2), which caused 20 cases resulting in three fatalities. Genome sequencing and analysis revealed that the two outbreak strains are related but distinct and did not recently evolve from a common ancestor (Rychli et al., 2014b). Both strains belong to serotype 1/2a, but to different sequence types (ST): QOC1 is assigned to ST403 and QOC2 to ST398.

*In vitro* and *in vivo* virulence assays showed that both strains are fully virulent. However, there were distinct differences in invasion efficiency and intracellular proliferation within different cell types: QOC1 was more virulent in non-phagocytic cells, whereas QOC2 showed higher invasion efficiency in phagocytic cells (Rychli et al., 2014b). The survival rate of mice orally infected with strain QOC2 was furthermore lower compared to strain QOC1. However, in this oral mouse infection model, QOC1 and QOC2 manifested a higher pathogenicity potential as compared to EGDe (Wagner et al., 2018). Regarding the virulence gene content, both outbreak strains have a functional *Listeria* pathogenicity island 1 (LIPI-1) and harbor the same set of eleven internalins (*inlA*, *inlB*, *inlC*, *inlC2*, *inlD*, *inlE*, *inlF*, *inlG*, *inlI*, *inlJ*, and *inlK*). Among all internalins two have been unambiguously linked to the attachment and internalization in non-phagocytic host cells: Internalin A (InlA) and Internalin B (InlB). InlA mediates the crossing of the primary entry site into the host, the intestinal epithelial barrier, upon binding to the respective eukaryotic cell surface receptor on epithelial cells, E-cadherin. InlB in turn enables *L. monocytogenes* to invade hepatocytes and various other cell types by interacting with the corresponding hepatocyte growth factor receptor, c-Met (Gaillard et al., 1991; Marino et al., 2000; Glaser et al., 2001; Kobe and Kajava, 2001; Cabanes et al., 2002; Bierne et al., 2007; Pizarro-Cerdá et al., 2012).

**FIGURE 1:**
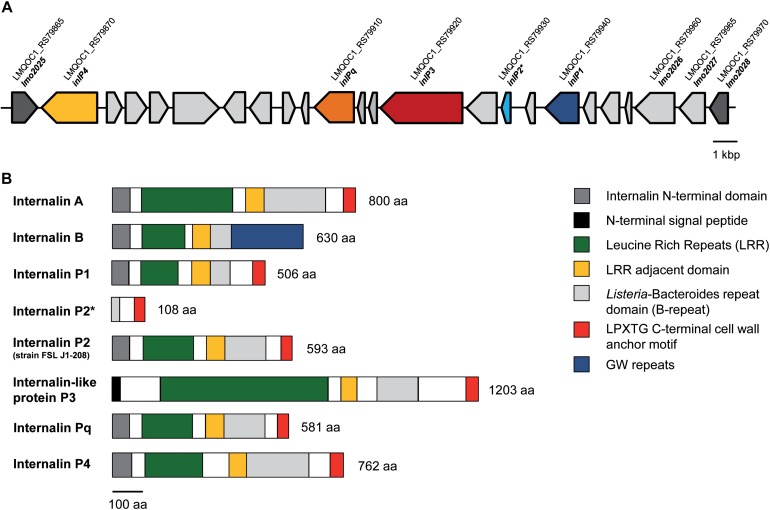
Genomic organization of the *lmo2025-lmo2028* region (hypervariable genetic hotspot 9) in *L. monocytogenes* QOC1. The QOC1 locus tags are indicated; dark gray: flanking genes of the hypervariable hotspot **(A)**. Comparative schematic protein domain organization of InlA, InlB, InlP1, the truncated InlP2, InlP3, InlPq, and InlP4 of *L. monocytogenes* QOC1 and the full length InlP2 of *L. monocytogenes* FSL J1-208. Homologous regions are color coded and legends are provided in boxes on the right. The numbers indicate the total protein length in amino acids (aa) **(B)**. ^*^ truncated.

Overall, QOC1 encodes 30 internalins or internalin-like proteins, while QOC2 encodes 26 such proteins. Four of these internalins or internalin-like proteins are only encoded by strain QOC1: *inlP1*, *inlP3*, *inlP4*, and one leucine-rich repeat domain protein (now named *inlPq*). They are located in an approximately 21 kbp long region between the *L. monocytogenes* EGDe homologous genes *lmo2025* and *lmo2028*, a locus that has recently been described as hypervariable genetic hotspot 9 in *L. monocytogenes* (Figure 1A; Kuenne et al., 2013; Rychli et al., 2014b). Apart from *inlP1*, *inlP3*, *inlPq* and *inlP4*, the pseudogene *inlP2*, a putative two-component regulatory system, ABC transporters, transposases, and other proteins of unknown function are encoded in this area (Rychli et al., 2014b).

Based on the similarities to other internalins, we hypothesized that the novel internalins InlP1, InlPq, and InlP4 and the internalin-like protein InlP3 have a role in the virulence and pathogenicity of *L. monocytogenes*. Therefore, we studied the expression of the four novel genes upon exposure to gastric stress and in intracellular bacteria in intestinal epithelial cells and assessed the role of the novel internalins and the internalin-like protein in an oral mouse infection model and subsequently in human intestinal epithelial cells and hepatocytes. We furthermore determined the prevalence of these genes by analyzing all available *L. monocytogenes* genome sequences.

## Materials and Methods

### *inlP4* Nucleotide Sequence

Since *inlP4* was present on two contigs (Rychli et al., 2014b), a gap-closing PCR was performed to determine the complete nucleotide sequence of *inlP4*. Therefore, two primer pairs targeting the flanking regions and the gap region between the two previously obtained contigs were used (Supplementary Table S1). The PCR products were purified and sequenced (LGC Genomics, Berlin, Germany) and the *inlP4* nucleotide sequence was determined by alignment using MAFFT (Katoh and Standley, 2013). The nucleotide sequence has been deposited in GenBank under the accession number MH800855.

### Protein Domain Organization of Internalins

Structural domains of InlA, InlB, InlP1, the truncated InlP2, InlP3, InlPq, and InlP4 of *L. monocytogenes* QOC1 and the full length InlP2 of strain FSL J1-208 were determined using the MotifFinder tool available on the GenomeNet website of the Kyoto University Bioinformatics Center^1^.

### Prevalence Study

Strains harboring *inlP1*, *inlP2*, *inlP3*, *inlPq*, and *inlP4* were retrieved from GenBank by BlastN using the NCBI genomes and whole genome shot gun contigs databases for *L. monocytogenes* (*n* = 2534, August 17, 2018) (Camacho et al., 2009). Determination of the ST of these strains was performed with the MLST tool available on the Center for Genomic Epidemiology website^2^ (Larsen et al., 2012). For each strain the nucleotide identity and sequence coverage of *inlP1*, *inlP2*, *inlP3*, *inlPq*, and *inlP4* were determined compared to the homologous genes of strain QOC1 using BlastN. To analyze whether *inlP1*, *inlP2*, *inlP3*, *inlPq*, and *inlP4* are located in the hypervariable genetic hotspot *lmo2025*-*lmo2028*, the location (the corresponding contigs) of the target genes and the genes *lmo2025* and *lmo2026* were determined using the NCBI genome database.

### Generation of *inlP1*, *inlP3*, *inlPq*, and *inlP4* Deletion Mutants and Complemented Strains

*inlP1*, *inlP3*, *inlPq*, and *inlP4* non-polar deletion mutants in *L. monocytogenes* QOC1 were generated using the splicing overlap extension PCR technique (SOEing-PCR) and the temperature-sensitive shuttle vector pKSV7 conferring chloramphenicol resistance were used according to Rychli et al. (2014a). For the complementation of the *inlP1*, *inlP3*, *inPq*, and *inlP4* mutant strains, the kanamycin-resistant, site-specific, integrative listeriophage vector pIMK2 (Monk et al., 2008) leading to constitutive gene over-expression was used. Details are described in the Supplementary Materials and Methods.

### Isolation of mRNA

For mRNA isolation, cells were disrupted using beadbeating in Lysing Matrix A tubes (MP Biomedicals) with a FastPrep FP120 instrument (MP Biomedicals). mRNA was subsequently isolated with TRIzol Reagent (Thermo Fisher Scientific) using chloroform phase separation and isopropanol RNA precipitation. Details are described in the Supplementary Materials and Methods.

### Quantitative RT-PCR

Primers targeting the *L. monocytogenes 16S rRNA*, *inlP1*, *inlP3*, *inlPq, inlP4*, and *inlA* genes were designed using Primer3 (v.0.4.0) (Supplementary Table S1). PCR conditions were applied based on Platinum Taq DNA Polymerase PCR set-up instructions: 1 × PCR Buffer, 250 nM primer forward and reverse, 3.75 mM MgCl_2_, 1.5 mM dNTP-Mix, 2 U Platinum Taq DNA polymerase (Life Technologies), and 1 μM EvaGreen (Jena Bioscience) in a final volume of 20 μl. 5 μl of cDNA template was used. Cycling conditions for *16S rRNA* were as follows: initial denaturation at 94 °C for 2 min, 45 cycles of denaturation at 94 °C for 30 s and annealing at 60 °C for 1 min, 3 cycles of denaturation at 95 °C for 1 min and annealing at 60 °C for 3 min, and final elongation at 95 °C for 30 s. Cycling conditions for *inlP1*, *inlP3*, *inlPq*, *inlP4*, and *inlA* were as follows: initial denaturation at 94 °C for 2 min, 45 cycles of denaturation at 94 °C for 30 s and annealing at 60 °C for 1 min, 3 cycles of denaturation at 95 °C for 1 min, and annealing at 60 °C for 30 s and final elongation at 95 °C for 30 s. All qRT-PCR reactions were performed using the Mx3000PTM cycler (Stratagene^®^). Subsequently, a dissociation curve was established (55–95 °C, 0.1 °C/second). A dilution series of genomic QOC1 wild type DNA (1–10^–6^ ng/μl) was used to determine the primer efficiency (1.86–1.99 for *16S rRNA*, 1.83–1.93 for *inlP1*, 1.98–1.99 for *inlP3*, 1.95–2.03 for *inlPq*, 1.85–1.89 for *inlP4*, and 1.94–1.99 for *inlA*). Data were analyzed using Mx3000PTM MxPro software Stratagene^®^. Each sample was measured in duplicates and relative quantification was performed using the comparative Ct method. Values, given as x-fold of QOC1 wild type control were normalized to *16S rRNA* as an internal reference (Tasara and Stephan, 2007). Mean values ± standard deviation (SD) of three biological replicates performed in triplicates and measured in duplicates were calculated.

### Protein Extraction, SDS-PAGE, and Western Blot Analysis

Cell wall proteins were isolated as previously described by Monk et al. (2004) with slight modifications. 10 μg of protein of each sample were loaded on an 8% sodium-dodecyl-sulfate-polyacrylamide gel. Semi dry blotting and subsequently a rabbit polyclonal anti-internalin A antibody (1:2000, cat. no. abx318926, Abbexa) and a secondary goat anti-rabbit-IgG antibody conjugated to horseradish peroxidase (1:2000, #7074, Cell Signaling) were used to visualize InlA surface exposure. Details are described in Supplementary Materials and Methods.

### Oral Mouse Infection Model

Mice were housed under specific pathogen-free conditions according to FELASA guidelines. All animal experiments were approved by the Ethics and Animal Welfare Committee of the University of Veterinary Medicine Vienna, conforming to the guidelines of the national authorities (BMWFW-68.205/0032-WF/II/3b/2014), FELASA and ARRIVE.

In order to evaluate an oral *L. monocytogenes* mouse infection model in advance, 8 weeks old female BALB/c mice (*n* = 18) were orally infected with 5 × 10^8^ CFU of QOC1 wild type strain resuspended in 200 μl Dulbecco’s Phosphate Buffered Saline (PBS; Thermo Fisher Scientific). Therefore, one colony of *L. monocytogenes* QOC1 was inoculated in 15 ml BHI-Y and cultivated for 8 h at 37°C shaking. Bacterial cultures were adjusted to an OD_600_ 0.1 in 100 ml BHI-Y and cultivated for 16 h at 37°C shaking. Bacteria were harvested by centrifugation for 5 min at 3220 *g*, washed twice with PBS and resuspended in PBS. Additionally, three animals were administered 200 μl PBS (control). Seven (six infected and one control) animals were anesthetized intraperitoneally using ketasol/xylasol and sacrificed by cervical dislocation 24, 48, and 72 h post infection. The liver, the spleen, and the small intestine were harvested and homogenized. The number of colony forming units (CFU) per organ was determined by plating serial dilutions of the liver and spleen homogenates on TSA-Y and the small intestine homogenates on TSA-Y and PALCAM agar (Becton Dickinson) in triplicate. The plates were incubated at 37°C for 48 h.

To further study the role of InlP1, InlP3, InlPq, and InlP4 during *L. monocytogenes* pathogenicity, 8 weeks old female BALB/c mice were orally infected with 5 × 10^8^ CFU of either QOC1 wild type, *inlP1*, *inlP3*, *inlPq*, or *inlP4* deletion mutant strain (*n* = 5–6 mice per strain). Bacteria were cultivated as described above. Additionally, two control animals were included (administered 200 μl PBS). The animals were anesthetized intraperitoneally using ketasol/xylasol, followed by retrobulbar blood sampling 72 h post infection and sacrificed by cervical dislocation. The liver, the spleen and the small intestine were harvested and homogenized. The number of CFU per organ was determined as described above.

### *In vitro* Virulence Assays

For the *in vitro* virulence assays, human intestinal epithelial Caco2 (ATCC^®^ HTB-37^TM^) and hepatocytic HEPG2 cells (ATCC^®^ HB-8065^TM^) were cultivated in Eagle’s minimum essential medium (MEM; Thermo Fisher Scientific) containing 2 mM L-glutamine, 10% fetal bovine serum (FBS), 100 units/ml penicillin, 100 mg/ml streptomycin sulfate and 0.25 mg/ml amphotericin B as well as 1% non-essential amino acids at 37°C in a humidified atmosphere (95% relative humidity) containing 5% CO_2_. Prior to infection, confluent cell monolayers (175 cm^2^) were washed twice with PBS, harvested by trypsinization (Trypsin-EDTA 0.25%) and seeded in 24-well plates in MEM containing 2 mM L-glutamine, 10% FBS, 100 units/ml penicillin, 100 mg/ml streptomycin sulfate, and 0.25 mg/ml amphotericin B as well as 1% non-essential amino acids. The medium was changed 16 h before infection to MEM containing 2 mM L-glutamine and 10% fetal bovine serum without antibiotics. Single colonies of the *L. monocytogenes* QOC1 wild type and the *inlP1*, *inlP3*, *inlPq*, and *inlP4* deletion mutant strains were inoculated in BHI-Y and the corresponding complemented mutant strains were inoculated in BHI-Y supplemented with 25 μg/ml kanamycin. Bacterial strains were cultivated for 16 h at 37°C shaking at 150 rpm. The bacterial cultures were adjusted to an OD_600_ 0.3 in 10 ml BHI-Y and incubated for 2 h at 37°C without shaking to reach late exponential/early stationary growth phase.

Cell monolayers were infected with *L. monocytogenes* at a multiplicity of infection of 25 for 1 h at 37°C. CFU of the inocula were determined by plating serial dilutions on TSA-Y. The infected cell monolayers were subsequently washed with PBS and incubated in MEM containing 10% FBS and 100 μg/ml gentamicin for 45 min (invasion) and 4 h (intracellular proliferation), respectively. After the incubation periods, the cells were lysed using 1 ml 0.1% Triton X-100 (Merck). CFU were determined by plating serial dilutions on TSA-Y. The invasion efficiencies were calculated as CFU recovered after 45 min of incubation in gentamicin-containing medium divided by the mean CFU of the inoculum. The intracellular growth coefficient was determined as follows: IGC = (intracellular bacteria _4__h_– intracellular bacteria _45__min_)/intracellular bacteria _45__min_. Each experiment was performed in triplicates and repeated three times.

### Statistical Analysis

Statistical analysis was performed using SPSS.20 software (SPSS Inc., Chicago, IL, United States). The mean values and SD were calculated. Brown Forsythe and Welch tests were applied to confirm variance homogeneity. Games-Howell posthoc test (variance heterogeneity) was used to compare the bacterial load in the small intestine, the liver and the spleen in orally infected mice with QOC1 wild type 24, 48, and 72 h post infection. *T*-test with independent variables (variance heterogeneity) was used to compare the gene expression of *inlP1*, *inlP3*, *inlPq*, and *inlP4* between the control and the gastric stress exposed or the intracellular bacteria (45 min and 4 h). Furthermore, *t*-test with independent variables (variance heterogeneity) was used to compare the bacterial load in the small intestine, the liver, and the spleen of mice infected with QOC1 wild type and the deletion mutant strains (variance homogeneity). To determine significant differences between the *inlA* expression, *in vitro* invasion efficiency and intracellular growth coefficient of QOC1 wild type, the deletion mutant strains and the corresponding complemented strains, a posthoc test (Games-Howell in the case of variance heterogeneity) was used. *p*-values < 0.05 were considered to be statistically significant.

## Results

### Protein Domain Characterization of InlP1, InlP2, InlP3, InlPq, and InlP4 of *L*. *monocytogenes* QOC1

The novel internalins InlP1, InlPq, and InlP4 harbor the representative N-terminal internalin signal sequence and leucine rich repeats (LRRs) in the amino terminal domain (Figure 1B), assigning them to the family of internalins, the pivotal virulence factors of *L. monocytogenes* (Bierne et al., 2007). InlP3 harbors LRRs (six LRR_8 domains) in the amino terminal domain in addition to a signal peptide different from the N-terminal internalin signal sequence and can therefore be assigned to the internalin-like proteins. InlP1 harbors two LRR_8 domains, InlP3 six (similar to InlA, which harbors six LRR_8 domains), InlPq three (similar to InlB, which harbors three LRR_8 domains) and InlP4 four. The LRRs of all four proteins are followed by a LRR adjacent domain and by *Listeria*-Bacteroides repeat domains (B-repeats). Furthermore, the carboxy-terminal domains of InlP1, InlP3, InlPq, and InlP4 harbor a LPXTG motif, which covalently anchors the proteins to the peptidoglycan in the cell wall, similarly, to InlA (Figure 1B; Braun et al., 1997; Bierne et al., 2007; Rajabian et al., 2009). InlP2 is not encoded in full length in *L. monocytogenes* QOC1 and lacks almost all the characteristic internalin domains due to a truncation but harbors B-repeats and a C-terminal LPXTG cell wall anchoring motif. The full length InlP2 encoded on the plasmid pLMIV of strain FSL J1-208 harbors the N-terminal internalin domain, LRRs (three LRR_8 domains), a LRR adjacent domain, B-repeats, and a C-terminal LPXTG sorting motif.

### Occurrence of *inlP1*, *inlP2*, *inlP3*, *inlPq*, and *inlP4*

The *in silico* prevalence study revealed that *inlP1*, *inlP2*, *inlP3*, *inlPq*, and *inlP4* are rarely present in *L. monocytogenes*. Only 15 strains harbored at least one of the target genes, among them five with human clinical origin, three from animal cases, five from food and one from the food processing environment (Table 1). The source of one strain was unknown. Eight strains harbored all five target genes and seven strains only *inlP4*. *inlP2*, present in eight strains, was truncated in six strains. All strains except two belonged to lineage II. The strains could be classified into eight different STs, belonging to seven clonal complexes (CC). Remarkably, 60% of the strains were attributed to CC19, among them seven of ST19 and two of ST378 (Table 1).

**TABLE 1:** Occurrence of *inlP1*, *inlP2*, *inlP3*, *inlPq*, and *inlP4.*

**#**	**Strain ID**	**GenBank accession number**	**Source**	**ST**	**CC**	**Lineage**	**Location**	***inlP1***	***inlP2***	***inlP3***	***inlPq***	***inlP4***
1	QOC1	CBVZ000000000.1	Human	403	403	II	Chromosome	+	+^*^	+	+	+
2	A522	MDQS00000000.1	Food	19	19	II	Chromosome	−	−	−	−	+
3	BCW_3917	MJNP00000000.1	Unknown	378	19	II	Chromosome	−	−	−	−	+
4	CFSAN046005	NXTP00000000.1	Smoked salmon	19	19	II	Chromosome	+	+^*^	+	+	+
5	FSL J1-208	CM001469.1	Caprine	569	ST569	IV	Plasmid	+	−	+	+	+
6	L2074	CP007689.1	Human	378	19	II	Chromosome	−	−	−	−	+
7	LM83088	CYWM00000000.1	Human	19	19	II	Chromosome	+	+^*^	+	+	+
8	LM-F-52	QABU00000000.1	Food	19	19	II	Chromosome	−	−	−	−	+
9	LM-F-82	QACN00000000.1	Food	19	19	II	Chromosome	−	−	−	−	+
10	LM-F-83	QACO00000000.1	Food	19	19	II	Chromosome	−	−	−	−	−
11	NRRL B-33014	MJOD00000000.1	Monkey	1065	ST1065	II	Chromosome	+	+^*^	+	+	+
12	NRRL B-33387	MKOC00000000.1	Bovine	556	ST556	II	Chromosome	+	+^*^	+	+	+
13	NRRL B-33568	MKOO00000000.1	Human	796	193	II	Chromosome	+	+^*^	+	+	+
14	OCF61	MDOY00000000.1	Food processing environment	19	19	II	Chromosome	−	−	−	−	+
15	SLCC2540	FR733645.1	Human	617	344	I	Chromosome	+	+	+	+	+

^*^ truncated.

The novel internalins showed a high similarity with a nucleotide identity from 91.74 to 99.86% for *inlP1*, from 94 to 100% for *inlP2*, from 96.35 to 99.49% for *inlPq* and from 81.96 to 99.91% for *inlP4* compared to the homologous genes in strain QOC1. In parallel *inlP3* showed a similarity from 96.19 to 99.75% (Supplementary Table S2). These genes were located on the chromosome in all strains except for strain FSL J1-208, where they were located on the plasmid pLMIV (den Bakker et al., 2012). Analysis of the exact location in the chromosome revealed that *inlP1*, *inlP2*, *inlP3*, *inlPq*, and *inlP4* are most probably located in the hypervariable genetic hotspot 9 in all strains (Supplementary Table S2).

### Gene Expression of *inlP1*, *inlP3*, *inlPq*, and *inlP4* Is Increased Under Gastric Stress and in Intracellular *L*. *monocytogenes*

**FIGURE 2:**
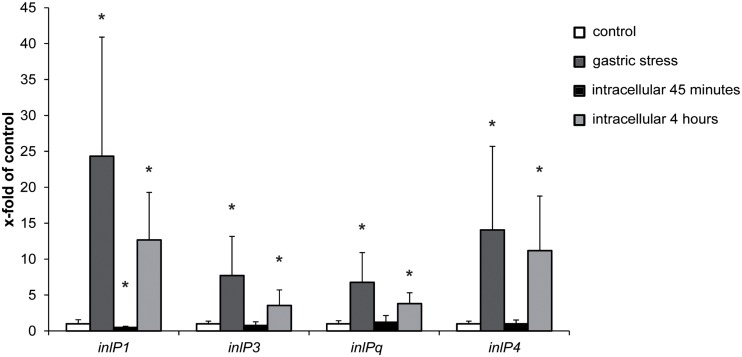
mRNA expression of *inlP1*, *inlP3*, *inlPq* and *inlP4* in *L. monocytogenes* QOC1 wild type strain incubated in defined minimal medium (control, white), synthetic gastric fluid for 30 min at 37°C (gastric stress; dark gray) and of intracellular bacteria in Caco2 cells 45 min (black), and 4 h (light gray) post infection (regarding the start of the gentamicin treatment). Values were normalized to *16S rRNA* expression levels and are presented as x-fold of the control. Data represent mean values ± SD of three biological replicates, performed and measured in triplicates. ^*^p < 0.05 vs. control.

In order to obtain first insights regarding the role of *inlP1*, *inlP3*, *inlPq*, and *inlP4* in the pathogenicity of *L. monocytogenes*, we assessed the gene expression in QOC1 wild type under *in vitro* conditions that mimic conditions *L. monocytogenes* encounters during infection of the human host: under gastric stress simulated by artificial gastric fluid and in intracellular bacteria in human intestinal epithelial Caco2 cells after 45 min and 4 h post infection. The expression of all four target genes was significantly increased after exposure to gastric fluid (Figure 2). Transcription of *inlP1* and *inlP4* was 24-fold and 14-fold higher under gastric stress conditions compared to the control, whereas the transcription of *inlP3* and *inlPq* increased 8- and 7-fold, respectively. In intracellular *L. monocytogenes* gene expression of all four genes was unchanged 45 min post infection except for *inlP1*, where a slight decrease was observed (Figure 2). In contrast, the transcription was significantly higher in intracellular bacteria 4 h after infection compared to the control (13-fold for *inlP1*, 11-fold for *inlP4*, fourfold for *inlP3*, and *inlPq*, Figure 2). In parallel, the expression of *inlA* in *L. monocytogenes* QOC1 wild type increased significantly after exposure to gastric fluid: 2.40-fold (*SD* = 0.51) compared to the control. Furthermore, we detected a strong induction of *inlA* gene expression in intracellular bacteria in Caco2 cells 45 min and 4 h after infection: 320.93-fold (*SD* = 95.05) after 45 min and 400.01-fold (*SD* = 82.64) after 4 h compared to the control, respectively.

These results propose a potential contribution of the novel internalins InlP1, InlPq, and InlP4 and the novel internalin-like protein InlP3 during the initial steps of human *L. monocytogenes* infection.

### InlP1 and InlP4 Contribute to the Colonization of the Spleen and the Liver in Orally Infected Mice

The analysis of the bacterial load in the small intestine, the liver and the spleen in orally infected mice with 5 × 10^8^ CFU of QOC1 wild type revealed detectable bacteria in the small intestine at 24, 48, and 72 h post infection and colonization of the liver and spleen 48 and 72 h post infection in all mice (Supplementary Figure S1). We observed a statistically significant difference in the bacterial load of all organs between the time points 24 and 72 h post infection with a significant decrease in the small intestine and a significant increase in the liver and the spleen (Supplementary Figure S1).

**FIGURE 3:**
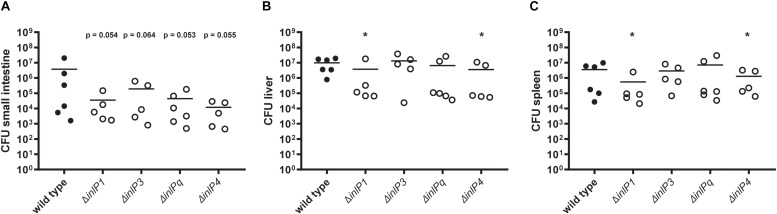
Colonization of the small intestine **(A)**, the liver **(B)**, and the spleen **(C)** of female BALB/c mice orally infected with 5 × 10^8^ CFU of *L. monocytogenes* QOC1 wild type and *inlP1*, *inlP3*, *inlPq*, and *inlP4* deletion mutant strains (*n* = 5–6 mice per strain). Total numbers of *L. monocytogenes* per organ were determined 72 h post infection. Data represent the mean CFU per animal. ^*^p < 0.05 vs. wild type.

Therefore, the role of the novel internalins and the internalin-like protein in the colonization of the target organs was determined 72 h post infection using the wild type and individual *inlP1*, *inlP3*, *inlPq*, and *inlP4* deletion mutant strains. We observed a distinct trend toward a decreased colonization of the small intestine in animals infected with one of the deletion mutant strains compared to the wild type (Figure 3A). However, the differences were not significant. The bacterial counts in the two major target organs for proliferation of *L. monocytogenes*, namely the liver and the spleen, were significantly reduced upon deletion of either *inlP1* or *inlP4* compared to the wild type (Figures 3B,C). Although the observed effects of the *inlP1* or *inlP4* deletion mutant strain were only small, these results suggest that InlP1 and InlP4 have a potential role in the pathogenicity of *L. monocytogenes* at least in the given experimental setting.

### InlP1 and InlP3 Influence the Invasion of *L*. *monocytogenes* in Human Intestinal Epithelial Cells

**FIGURE 4:**
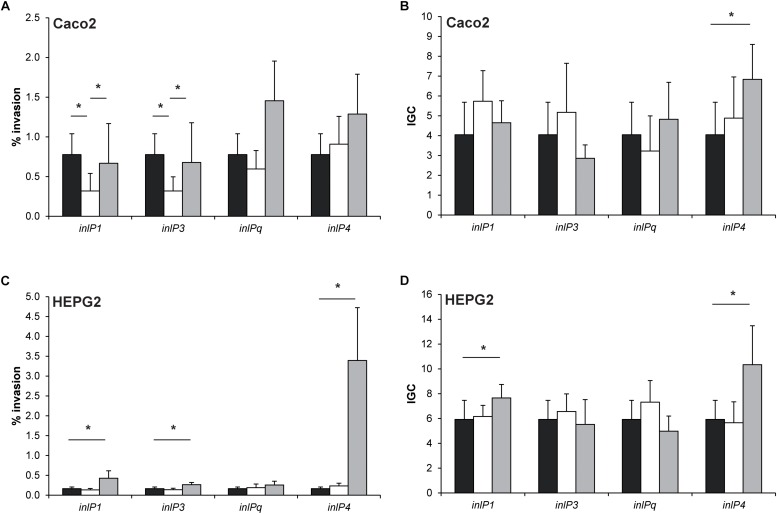
Invasion efficiency **(A**,**C)** and intracellular growth coefficient (IGC, **B**,**D**) of *L. monocytogenes* QOC1 wild type (black bars) and *inlP1*, *inlP3*, *inlPq*, and *inlP4* deletion mutant strains (white bars) and complemented strains (gray bars) were evaluated in the human intestinal epithelial cells Caco2 **(A**,**B)** and hepatocytic HEPG2 **(C**,**D)** cells. Data represent mean values ± SD of three biological replicates performed in triplicates. ^*^p < 0.05.

To determine whether the three novel internalins and the internalin-like protein affect the invasion efficiency and intracellular proliferation in human cells, *in vitro* virulence assays were performed in human intestinal epithelial Caco2 cells and HEPG2 hepatocytes using the wild type, the deletion mutants and the corresponding complemented strains (Figure 4). As compared to the wild type, the complementation of the respective deletion mutant strains resulted in an increase of gene expression: 145.96-fold (*SD* = 42.47) of *inlP1*, 130.09-fold (*SD* = 46.56) of *inlP3*, 375.29-fold (*SD* = 7.82) of *inlPq* and 7.26-fold (*SD* = 5.62) of *inlP4*.

In Caco2 cells, the invasion efficiencies of the *inlP1* and the *inlP3* deletion mutant strain were significantly lower compared to the wild type. These phenotypes could be reversed in the respective complemented strains (Figure 4A). The intracellular replication in Caco2 cells was similar in all strains with one exception. The complemented *inlP4* deletion mutant strain showed significantly higher intracellular proliferation in Caco2 cells compared to the wild type strain (Figure 4B). To exclude that the observed effects were due to altered *inlA* expression we analyzed the gene expression of *inlA* in the wild type strain, the deletion mutants and the complemented strains. No significant differences in the *inlA* expression were observed between all strains. In addition, we observed a uniform cell surface exposure of InlA in all strains at approximately equal amounts (Supplementary Figure S2).

The complemented *inlP1*, *inlP3* and *inlP4* mutant strain showed significantly enhanced invasion efficiencies in HEPG2 cells, whereas separate deletion of the four internalins showed no effect (Figure 4C). Additionally, we observed significantly higher intracellular growth coefficients in the complemented *inlP1* and the complemented *inlP4* strain (Figure 4D).

These results suggest that at least InlP1 and InlP3 contribute to invasion of human intestinal epithelial cells.

## Discussion

In this study, we characterized the pathogenic potential of the novel internalins InlP1, InlPq, and InlP4 and the novel internalin-like protein InlP3 in *L. monocytogenes* QOC1, one of two Austrian Quargel clones responsible for the multinational listeriosis outbreak in 2009/2010. QOC1 caused 14 severe listeriosis cases resulting in five fatalities, which corresponds to an exceptionally high mortality rate of 35.71% (Fretz et al., 2010a).

The protein domain characterization demonstrated that InlP1, InlPq, and InlP4 are equipped with conserved inherent domains of the protein family of internalins and have a similar structure like InlA. The N-termini of InlP1, InlPq, and InlP4 harbor an internalin domain, which is approximately 60 amino acids long and presumably caps the hydrophobic core and serves as a signal sequence in all known internalins (Schubert et al., 2002; Bierne et al., 2007). InlP3 lacks the internalin domain but harbors a signal peptide in the N terminus. Due to the absence of this particular domain in InlP3, we assigned it to the family of internalin-like proteins. All novel internalins and the novel internalin-like protein possess LRRs, the major property of internalins involved in protein-protein interactions, a LRR adjacent domain followed by a *Listeria*-Bacteroides repeat domain (B-repeat) and a LPXTG sorting motif at the C-termini (Bierne et al., 2007; Ebbes et al., 2011). The C-termini are completed by a domain of hydrophobic amino acids and positively charged residues after the LPXTG motif and thereby confer a covalent anchor to the peptidoglycan (Bierne et al., 2007). The truncated form of InlP2 does not show characteristics of internalins due to an internal truncation in the protein sequence with the exception of B-repeats and the LPXTG C-terminal cell wall anchor motif. However, the full length homolog encoded only by two strains (strain FSL J1-208 and SLCC2540) presents the N-terminal internalin domain, LRRs (three LRR_8 domains), a LRR adjacent domain, B-repeats, and a C-terminal LPXTG sorting motif.

Our *in silico* prevalence study revealed that *inlP1*, *inlP2*, *inlP3*, *inlPq*, and *inlP4* rarely occur in *L. monocytogenes*. Only eight out of 2534 strains, whose genome was available, were identified to harbor all five target sequences. Additional seven strains, all belonging to CC19, harbor only *inlP4*. Interestingly, 60% of the positive strains were assigned to CC19, including seven ST19 and two ST378 strains. The CC19 strains were isolated between 1991 and 2013 in Italy, France, Canada, and the United States from different sources including food, the food processing environment and human clinical samples. Strains of CC19 belong to intermediate clones, meaning that they are neither explicitly associated with a clinical nor with a food origin and they are known to occur very rarely with a prevalence of 0.17–0.26%. In parallel, the CCs (and STs) of the other positive strains have been reported to also have very low prevalence (Maury et al., 2016; Nielsen et al., 2017). This indicates that only minor CCs harbor this accessory gene content located in the hypervariable genetic hotspot 9 in all strains with one exception (Kuenne et al., 2013): strain FSL J1-208 encodes the genes on the plasmid pLMIV (den Bakker et al., 2012). At least nine different inserts consisting of one to 20 genes are located in the hypervariable genetic hotspot 9 described to predominantly accommodate variations of surface-associated genes potentially involved in virulence (Kuenne et al., 2013).

We demonstrated that the expression of *inlP1*, *inlP3*, *inlPq*, and *inlP4*, especially *inlP1* and *inlP4*, increased significantly upon exposure to gastric stress and in intracellular bacteria grown in human intestinal epithelial cells for 4 h. This suggests that these genes are potentially activated during initial host stress conditions and intracellular proliferation in the primary entry site at human body temperature, i.e., 37°C. We additionally confirmed that *inlA* expression was increased following exposure to gastric stress and in intracellular *L. monocytogenes* in infected human intestinal epithelial cells after 45 min and 4 h. This is in accordance with previous studies showing that not only the expression of *inlA* but also of *inlB* and *inlC* were increased in intracellular *L. monocytogenes* in murine macrophages and human intestinal epithelial Caco2 cells, thereby underlining the importance of these internalins during the infectious process in the host cell cytosol (Chatterjee et al., 2006; Joseph et al., 2006).

To investigate the role of InlP1, InlP3, InlPq, and InlP4 in the pathogenesis *in vivo*, we used an oral mouse infection model that allows a close imitation of the natural transmission route and the host environment including gastric stress despite the higher deviation in CFU counts that accompanies oral infection. The CFU deviation is due to individual responses in the mice in comparison to intravenous infection based on the fact that orally acquired bacteria have to pass a series of bottlenecks to reach target organs for colonization (Pitts and D’Orazio, 2018). We explicitly did not use an intravenous mouse infection model because listeriosis is not initiated by high bacterial loads in the blood and the colonization of peripheral tissues can be affected by preliminary colonization of the gut which is entirely avoided when *L. monocytogenes* is applied intravenously (Pitts and D’Orazio, 2018). In general, oral *L. monocytogenes* mouse infection models distort the species-specific invasion process of the primary entry site that relies on the interaction between InlA, the most important virulence factor during invasion, and E-cadherin, the corresponding receptor on epithelial cells. Despite the lack of the interaction between InlA and mouse E-cadherin due to a structural deviation (Lecuit et al., 1999), we and others proved that colonization of target organs was still effective in an oral mouse infection model using *L. monocytogenes* (Barbour et al., 2001; Bou Ghanem et al., 2012; Feehily et al., 2014; Jones et al., 2015). The colonization of the small intestine in the oral mouse infection model might be attributed to paracellular routes. Transcytosis through microfold (M) cells present in the Peyer’s patches has been described to be InlA- and InlB-independent, both *in vitro* in a human Caco2 cell monolayer as well as *in vivo* in an oral mouse infection model. M-cell transcytosis permits *L. monocytogenes* to rapidly access mucosal lymphoid tissue and thereby enables systemic spread (Corr et al., 2006). More recently, *Listeria* adhesion protein (LAP) was reported to mediate translocation through Caco2 cells *in vitro* and into the lamina propria of the small intestine of orally infected mice in an InlA-independent way by increasing epithelial permeability (Drolia et al., 2018).

Since colonization of the small intestine, the liver and the spleen by orally acquired bacteria might take up to 48 h and can easily appear in asynchronous phases (Pitts and D’Orazio, 2018), we first determined the colonization efficiency at three time points in our experimental setting. Bacteria were detected in the small intestine 24, 48, and 72 h post infection with a significant decrease between 24 and 72 h. In contrast, sufficient colonization of the liver and spleen, the major target organs of *L. monocytogenes* replication, was detected only after 48 and 72 h in all animals, with a significant increase between 24 and 72 h. These time-dependent observations are in line with the innate infection route: *L. monocytogenes* encounters the small intestine as the primary entry site in the host where colonization occurs initially and decreases as the pathogen is shed via feces. Subsequently, *L. monocytogenes* spreads via the lymph and the blood circulation to peripheral organs where the colonization successively increases.

To test the role of the novel internalins and the internalin-like protein on the pathogenicity of *L. monocytogenes* we mainly studied the colonization of the target organs of replication – the liver and the spleen. Deletion of *inlP1* or *inlP4* furthermore resulted in a significantly lower bacterial burden in the liver and the spleen. Although the observed effects were small, this suggests that InlP1 and InlP4 have a potential role in contributing to the pathogenicity of *L. monocytogenes* at least in the given experimental setting. These observations nicely correlate with the *inlP1* and *inlP4* gene expression patterns, which were the highest among all four novel genes *in vitro* after exposure to gastric stress and in intracellular bacteria after 4 h of proliferation.

To complement the obtained *in vivo* results from the mouse model, we determined the virulence potential of QOC1 wild type, the deletion mutants, and the corresponding complemented strains *in vitro* in intestinal epithelial Caco2 cells, in which the E-cadherin-InlA interaction is functional, and HEPG2 hepatocytes. These cell lines perfectly represent both the primary entry site of *L. monocytogenes* in the human host, the intestinal epithelium, and one of the major target organs of replication, the liver. The second target organ of replication, the spleen, consists of a consortium of different cell populations such as lymphocytes, monocytes, and dendritic cells comprising roughly 90% of splenocytes. Because these cell types do not efficiently serve as intracellular niches for *L. monocytogenes*, the majority of the bacterial load in the spleen is considered to be extracellular (McElroy et al., 2009; Jones and D’Orazio, 2017). Although splenic cell types are individually available as cell lines in suspension, they do not represent the entire organ organization and are not suitable for modeling *L. monocytogenes* invasion and intracellular proliferation.

We showed that upon deletion of *inlP1* or *inlP3* invasion was significantly lower in intestinal epithelial cells as compared to the wild type and that this effect could be reversed when the respective gene was complemented. These results indicate that InlP1 and InlP3 only contribute to the invasion in intestinal epithelial cells as the process was not entirely impaired using the deletion mutants. Since *inlA* expression as well as InlA cell surface exposure was not altered in the deletion mutant strains and the complemented strains in comparison to the wild type, we can exclude an underlying effect of InlA. These data are in contrast to the study of den Bakker et al. (2012) in which no difference regarding the invasion efficiency of human intestinal epithelial Caco2 cells between the parental *L. monocytogenes* FSL J1-208 strain harboring the pLMIV plasmid encoding the novel internalin homologs and the plasmid-cured strain were observed.

Over-expression of specific genes derived from the complementation using a constitutive expression vector resulted in induced *in vitro* virulence, e.g., induced expression of *inlP4* increases intracellular growth in Caco2 cells and invasion and intracellular growth in HEPG2 cells. We can only speculate why we detected differences in the complemented strains and not in the corresponding deletion mutant strains. One reason might be the high over-expression detected in most of the complemented strains.

The divergences between the *in vivo* and *in vitro* results might be attributed to differences in internalin-receptor interactions between mice and human cell lines on the one hand and to the different time points monitoring the bacterial load inside the target organs and the cells on the other hand. From this point of investigation, we cannot exclude that over-expression of the respective internalins and the internalin-like protein might have a similar effect *in vivo* as it displayed *in vitro*.

In conclusion, this study reports the contribution of the uncommon novel internalins InlP1, InlPq, and InlP4 and the internalin-like protein InlP3 to the virulence potential of *L. monocytogenes* QOC1. All four genes are transcriptionally activated after exposure to gastric stress and in intracellular bacteria. Despite their overall low abundance they are certainly co-responsible among other virulence factors for the pathogenicity of *L. monocytogenes* QOC1.

## Data Availability

The datasets generated for this study can be found in the GenBank, MH800855.

## Ethics Statement

The mice used in this study were housed under specific pathogen-free conditions according to the FELASA guidelines. All animal experiments were approved by the Ethics and Animal Welfare Committee of the University of Veterinary Medicine Vienna, conforming to the guidelines of the national authorities (BMWFW-68.205/0032-WF/II/3b/2014), FELASA, and ARRIVE.

## Author Contributions

EH, CL, MW, and KR were involved in the design of the study and interpretation of the data. EH, CL, EW, and AZ were involved in the acquisition and analysis of the data. EH and KR wrote the manuscript. All authors contributed to the manuscript revision, and read and approved its submitted version.

## Conflict of Interest Statement

The authors declare that the research was conducted in the absence of any commercial or financial relationships that could be construed as a potential conflict of interest.

## Footnotes

1 https://www.genome.jp/tools/motif/

2 https://cge.cbs.dtu.dk/services/MLST/
