# Supplementary material for: The Novel Internalins InlP1 and InlP4 and the Internalin-Like Protein InlP3 Enhance the Pathogenicity of Listeria monocytogenes
Source: Front Microbiol. 2019 Jul 23;10:1644. doi: 10.3389/fmicb.2019.01644 (PMC6664051; doi:10.3389/fmicb.2019.01644)
Supplement: Supplementary file 1 [file Data_Sheet_1.PDF]

## Supplementary Materials and Methods

### Generation of *inlP1, inlP3, inlPq* and *inlP4* deletion mutants and complemented strains

Primers were designed (SoeA-D) to amplify two DNA segments (SoeAB and SoeCD) flanking the genomic region targeted for deletion and for the screening PCR (Table S1). PCR reactions were performed using Phusion Green High Fidelity DNA Polymerase (Fisher Scientific).

DNA was amplified from *L. monocytogenes* QOC1 wild type using Phusion Green High Fidelity DNA Polymerase and specific primer pairs (Table S1). The vector pIMK2 and the *inlP1*, *inlP3*, *inlPq* and *inlP4* purified PCR products were digested with two restriction enzymes (*inlP1*, *inlP3* and *inlP4*: *NcoI* and *PstI*; *inlPq*: *NcoI* and *BamHI*), ligated using T4 DNA ligase (Fisher Scientific) and transformed into competent *Escherichia coli* (StrataClone SoloPack, Agilent Technologies). The plasmid containing the respective gene of interest was then electroporated into competent QOC1 *inlP1*, *inlP3*, *inlPq* or *inlP4* deletion mutant strains. Positive transformants were selected on tryptic soy agar supplemented with yeast (TSA-Y; Merck) and 25 µg/ml kanamycin and confirmed by PCR.

### Protein extraction, SDS-PAGE and Western Blot analysis

Cell wall proteins were isolated as previously described by Monk et al. (Monk et al., 2004) with slight modifications. Single colonies of the *L. monocytogenes* QOC1 wild type and the *inlP1*, *inlP3*, *inlPq* and *inlP4* deletion mutant strains were inoculated in BHI-Y. The corresponding complemented mutant strains were inoculated in BHI-Y supplemented with 25 µg/ml kanamycin. Bacterial strains were cultivated for 16 hours at 37 °C shaking at 150 rpm. The bacterial cultures were adjusted to an $OD_{600}$ 0.3 in 10 ml BHI-Y and incubated for 2 hours at 37 °C without shaking to reach late exponential/early stationary growth phase. Bacteria were harvested by centrifugation at 3220 g for 10 minutes at room temperature. Each pellet was resuspended in 1 ml wash buffer (10 mM Tris-HCl, pH 6.9, 10 mM $MgCl_2$) and centrifuged at 7000 g for 10 minutes at room temperature. The supernatant was discarded and the pellet was resuspended in 1 ml wash buffer supplemented with 0.5 M sucrose and centrifuged at 7000 g for 10 minutes at room temperature. The supernatant was discarded and an additional centrifugation step was conducted to remove last traces of the supernatant at 7000 g for 2 minutes. The supernatant was discarded and the pellet was resuspended in 200 µl wash buffer supplemented with 0.5 M sucrose, 10 mg/ml lysozyme (lysozyme from chicken egg white; Sigma Aldrich), 250 U/ml mutanolysin (*Streptomyces globisporus* ATCC 21553; Sigma Aldrich) and 10 mM phenylmethanesulfonyl fluoride (Sigma Aldrich) as a protease inhibitor and incubated at 37 °C in a thermoblock shaking at 400 rpm. The lysate was centrifuged at 15000 g for 10 minutes and the supernatant containing the cell wall proteins was retained. Protein concentrations were quantified using the Qubit® Protein Assay Kit and the Qubit® 2.0 Fluorometer (Invitrogen). Amounts of 30 µg protein per sample were mixed with equal volumes of 2x Laemmli Sample Buffer and boiled for 5 minutes at 95 °C. 10 µg of protein of each sample were loaded on an 8 % sodium-dodecyl-sulfate-polyacrylamide gel. After electrophoresis (1 hour at constant 150 V) the proteins were transferred to a nitrocellulose membrane using a semi-dry blotting system (Trans-Blot SD, Biorad). The membrane was washed with 1x PY-TBST (10 mM Tris-HCl pH 7.4, 75 mM NaCl, 1 mM EDTA, pH 8, 0.1 % Tween 20), subjected to 1 hour of blocking with 5 % non-fat milk in 1x PY-TBST and probed with a primary rabbit polyclonal anti-internalin A antibody (1:2000, cat. no. abx318926, Abbexa) in 1 % bovine serum albumin (BSA) in 1x PY-TBST supplemented with 0.01 % sodium azide over night at 4 °C. The membrane was washed with 1x PY-TBST and incubated with a secondary goat anti-rabbit-IgG antibody conjugated to horseradish peroxidase (1:2000, #7074, Cell Signaling) in 1 % BSA in 1x PY-TBST for 1 hour. The membrane was washed again with 1x PY-TBST and antigen-specific binding of the

antibody was visualized using a chemiluminescence detection system (ECL Clarity Western and ChemiDoc Touch, Biorad).

**Isolation of mRNA**

A single colony of *L. monocytogenes* strain QOC1 wild type was inoculated in brain heart infusion supplemented with yeast (BHI-Y; Merck) in triplicates and cultivated overnight shaking at 37 °C. Each bacterial culture was adjusted to an optical density at 600 nm ($OD_{600}$) 0.2 three times: twice in 15 ml defined minimal medium (DMM; RPMI1640 supplemented with 0.088 g/l ferric(III)citrate) as control and for infection of Caco2 cells and once in synthetic gastric fluid (gastric stress) according to Cotter et al. (Cotter et al., 2001). Cultures were incubated for 30 minutes (control and gastric stress) or 2 hours (for infection of Caco2 cells) at 37 °C without shaking. After 30 minutes, control bacterial cultures and those exposed to gastric stress were centrifuged at 3220 g for 10 minutes at room temperature and the pellets were resuspended in 1 ml RNAlater® solution.

After 2 hours of incubation at 37 °C, bacterial cultures were harvested by centrifugation at 3220 g for 10 minutes and resuspended in Eagle's minimum essential medium (MEM; Fisher Scientific). To study gene expression of intracellular bacteria, monolayers (225 $cm^2$) of human intestinal epithelial Caco2 cells (ATCC® HTB-37™) were infected with *L. monocytogenes* QOC1 wild type in MEM at a multiplicity of infection of 25 for 1 hour at 37 °C. The infected cells were then washed with Dulbecco's Phosphate Buffered Saline (PBS; Fisher Scientific) and incubated in MEM containing 10 % fetal bovine serum (FBS) and 100 µg/ml gentamicin. After 45 minutes and 4 hours cells were harvested by trypsinization (Trypsin-EDTA 0.25 %; Fisher Scientific), centrifuged for 10 minutes at 3220 g at room temperature and the pellet was resuspended in 1.5 ml RNAlater® solution.

The over-expression of the novel internalins and the internalin-like protein was quantified in the corresponding complemented strains. Furthermore, the expression of *inlA* was quantified in the wild type, the deletion mutant strains and complemented strains under conditions used for *in vitro* virulence assays. Therefore, bacterial strains were cultivated in 8 ml BHI-Y or BHI-Y supplemented with 25 µg/ml kanamycin (complemented strains) for 16 hours at 37 °C shaking at 150 rpm. The bacterial cultures were adjusted to $OD_{600}$ 0.3 in 10 ml BHI-Y and incubated for 2 hours at 37 °C without shaking to reach late exponential/early stationary growth phase.

For RNA isolation, the bacteria were centrifuged at 3220 g for 5 minutes and resuspended in 1 ml TRIzol Reagent (Fisher Scientific). Cells were disrupted using beadbeating in Lysing Matrix A tubes (MP Biomedicals) with a FastPrep FP120 instrument (MP Biomedicals) and the following parameters: three times 45 seconds at speed 5.5 at 4 °C. RNA was isolated by chloroform phase separation and isopropanol RNA precipitation. The RNA pellet was washed with 75 % ethanol, dried and dissolved in 50 µl RNase free water. The RNA amount was measured using Qubit® 2.0 Fluorometer (Invitrogen). The remaining DNA was digested using the Turbo DNA-free Kit (Life Technologies, Agilent) according to manufacturer's instructions. A PCR targeting the *16S rRNA* gene was performed using DreamTaq DNA Polymerase Mix (Fisher Scientific) to confirm the absence of DNA. PCR cycling conditions were as follows: initial denaturation at 95 °C for 5 minutes; 30 cycles of denaturation at 95 °C for 15 seconds, annealing at 60 °C for 30 seconds, elongation at 72 °C for 30 seconds; final elongation at 72 °C for 2 minutes; hold at 4 °C. RNA amounts between 25 ng (for pure bacteria cultures) and 300 ng (for intracellular bacteria) were used for cDNA synthesis using the RevertAid H Minus First Strand cDNA Synthesis Kit (Thermo Scientific) according to the manufacturer's protocol.
