# Supplementary figures and images for: The Novel Internalins InlP1 and InlP4 and the Internalin-Like Protein InlP3 Enhance the Pathogenicity of Listeria monocytogenes
Source: Front Microbiol. 2019 Jul 23;10:1644. doi: 10.3389/fmicb.2019.01644 (PMC6664051; doi:10.3389/fmicb.2019.01644)

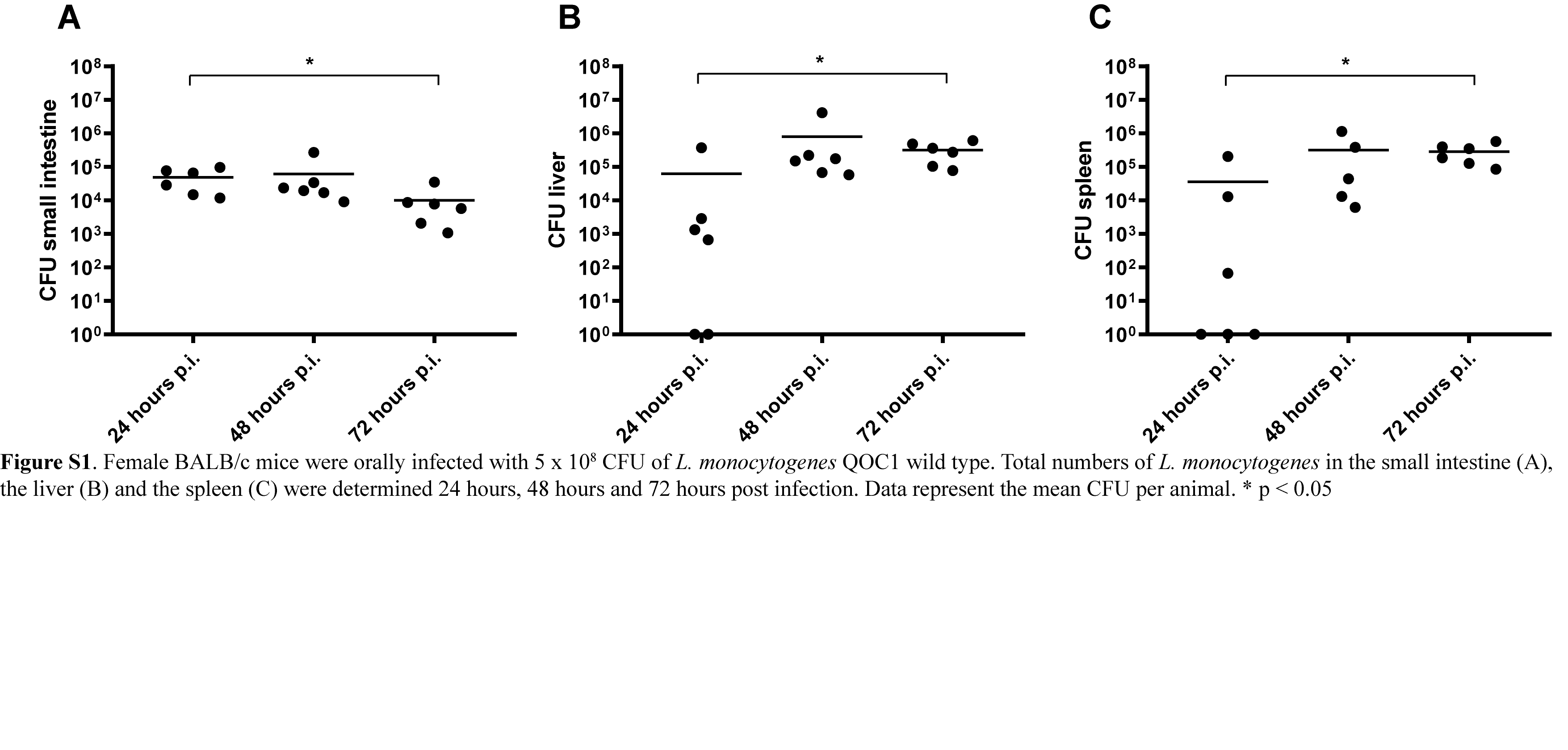

Supplement: Supplementary file 2 [file Image_1.TIF]

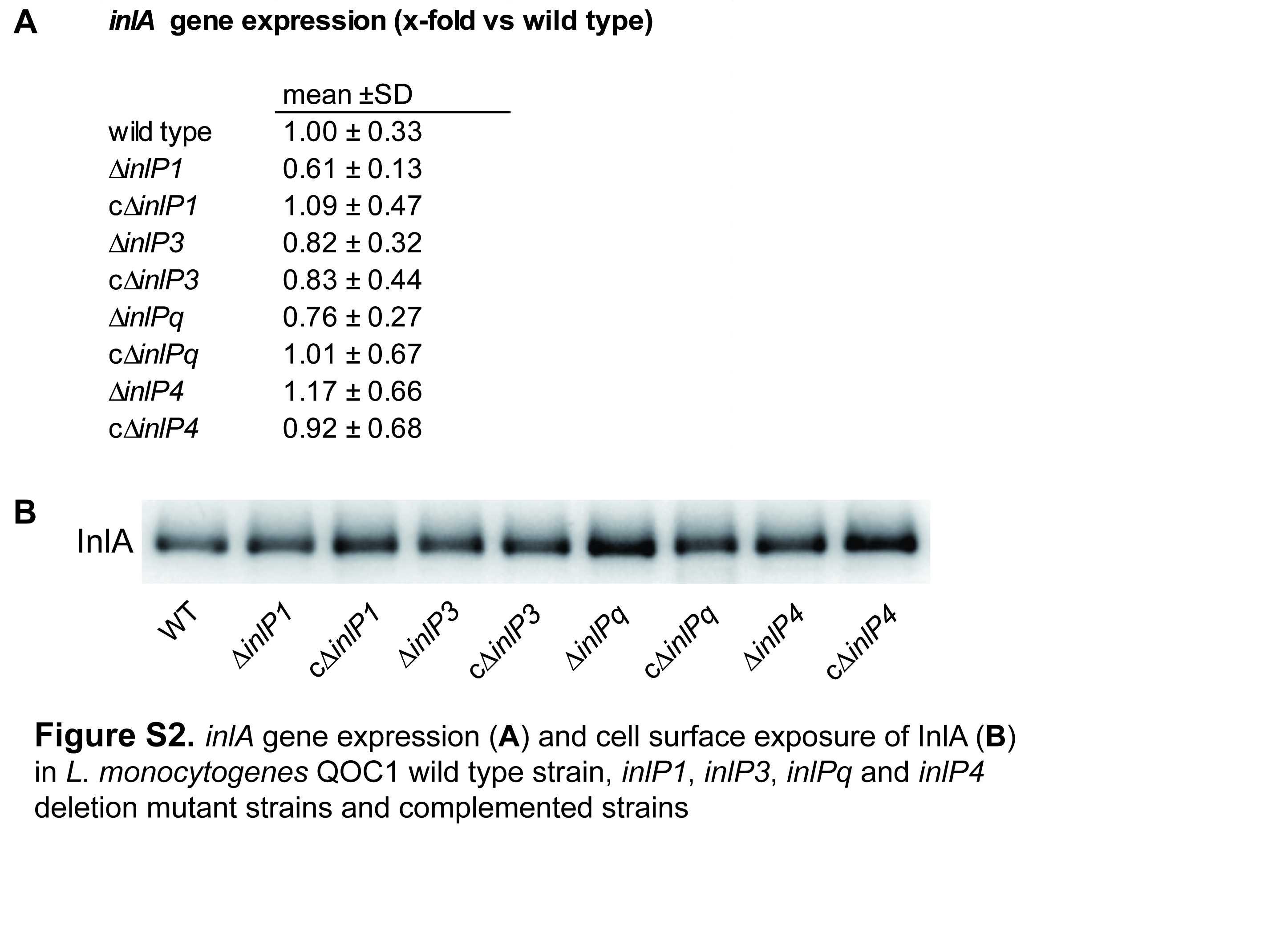

Supplement: Supplementary file 3 [file Image_2.TIF]
