# Supplementary material for: The Novel Internalins InlP1 and InlP4 and the Internalin-Like Protein InlP3 Enhance the Pathogenicity of Listeria monocytogenes
Source: Front Microbiol. 2019 Jul 23;10:1644. doi: 10.3389/fmicb.2019.01644 (PMC6664051; doi:10.3389/fmicb.2019.01644)
Supplement: Supplementary file 4 [file Table_1.DOCX]

**Table S1** Primers used in this study.

| **gene target** | **method** | **primer** | **sequence (5’-3’)** |
| --- | --- | --- | --- |
| *inlP4* | gap-closing PCR | forward 1 | AAGGAGCCGGAGGAAAAGACG |
|  |  | forward 2 | ATGGGTACTGGATGTAAGGC |
|  |  | reverse 1 | ACTATGATCCCCGGTTGCTGGTA |
|  |  | reverse 2 | ATAACTTACAGGTACTTCGAT |
| *16S rRNA* | qRT-PCR | forward | TTAGCTAGTTGGTAGGGTAATGGC |
|  |  | reverse | CAGTACTTTACGATCCGAAAACCT |
| *inlP1* | qRT-PCR | forward | TTTTATGCCGATGATGGGTGTTTCTA |
|  |  | reverse | AAGGTATTGGCATTATCTCTGACGGTATT |
| *inlP3* | qRT-PCR | forward | ACCAGCGTTTCCCTCTAGTTCCAGTAT |
|  |  | reverse | GGCAAATTCTCTAACCCGTCTAAGTCAG |
| *inlPq* | qRT-PCR | forward | AGGTGCGACGGGGACTTTCA |
|  |  | reverse | TCACTTGTTTCCACCCCGTCTAC |
| *inlP4* | qRT-PCR | forward | AAGGAGCCGGAGGAAAAGACG |
|  |  | reverse | ACTATGATCCCCGGTTGCTGGTA |
| *inlA* | qRT-PCR | forward | GCTGTACGCTCAATTCACGA |
|  |  | reverse | ATTTGCGGAAGGTGGTGTAG |
| *inlP1* | generation of deletion mutant | SoeA | ATGGAATTCAATTGCCAATTATGTG |
|  |  | SoeB | TAGTAATCTACCTCTTTCTCTCAG |
|  |  | SoeC | CTGAGAGAAAGAGGTAGATTACTAGCTACATGTATTCCAAGATTTG |
|  |  | SoeD | AGGCTGCAGCTCCCGCATACTGTT |
|  |  | SoeE | GTCACAACAAATCCTATCAATACC |
|  |  | SoeZ | CTTCATATCTATGGAATCTTACGT |
| *inlP3* | generation of deletion mutant | SoeA | AGGTCTAGAAATATGTAAACCATT |
|  |  | SoeB | GATTGGCCTCCTAGTTGATATGGA |
|  |  | SoeC | TCCATATCAACTAGGAGGCCAATCGAGTTTTTAGATGAGGAAAATTC |
|  |  | SoeD | AGGCTGCAGTTGTTGCCATACC |
|  |  | SoeE | CCATTTATAAAGTTCCCAACGTA |
|  |  | SoeZ | GGATCAAAATAATAATTAACAAGC |
| *inlPq* | generation of deletion mutant | SoeA | ATGGAATTCTTGTAGCATTATATT |
|  |  | SoeB | CTTAAATCTCCTTCCTTTTGATTT |
|  |  | SoeC | AAATCAAAAGGAAGGAGATTTAAGTAGCTAATAAGCTTAAGAAA |
|  |  | SoeD | AGGCTGCAGCTTCCTATCAACTGATTC |
|  |  | SoeE | AGATTATTCTTAATTCTTTTGAG |
|  |  | SoeZ | catatggctagagcagaatacgc |
| *inlP4* | generation of deletion mutant | SoeA | ATGGAATTCAAGCAAAGACAACT |
|  |  | SoeB | AGTTAGTAACTAAAAAATGTATC |
|  |  | SoeC | gatacattttttagttactaactgccacttgtataaattattttc |
|  |  | SoeD | AGGCTGCAGTCAGCTCCATAATT |
|  |  | SoeE | ACTAGAAGCTATCCAAAACGG |
|  |  | SoeZ | TAATTGCAGTTCGTGCCTTAT |
| *inlP1* | complementation | forward | CGGCCATGGAAGCTAAGAAATATCTCCA |
|  |  | reverse | CGGTCTAGACTCCCCTTTGTTGTACAG |
| *inlP3* | complementation | forward | CGGCCATGGAAGGAAAATTAAAATTTATC |
|  |  | reverse | CGGTCTAGAATCCCATTCGTAAGTTA |
| *inlPq* | complementation | forward | CGGCCATGGAAGATAAGAAACAATTC |
|  |  | reverse | CGGTCTAGAGACTACATTCATCCTCT |
| *inlP4* | complementation | forward | CGGCCATGGGAGAAAGAGGTAGATTAC |
|  |  | reverse | CGGTCTAGAGAAAATAATTTATACAAGTGG |
